# Cytotoxic Constituents from the Stems of *Clausena lansium* (Lour.) Skeels 

**DOI:** 10.3390/molecules180910768

**Published:** 2013-09-03

**Authors:** Hai Yan Jiang, Cheng Fang Wang, Li Fan, Kai Yang, Jiang Bin Feng, Zhu Feng Geng, Jing Xu, Zhi Wei Deng, Shu Shan Du, Hai Bo Yin

**Affiliations:** 1 College of Pharmacy, Liaoning University of Traditional Chinese Medicine, Dalian 116600, Liaoning, China; E-Mails: jhy880527@sina.cn (H.Y.J.); xujing020513@163.com (J.X.); 2 State Key Laboratory of Earth Surface Processes and Resource Ecology, Beijing Normal University, Beijing 100875, China; E-Mails: wangchengfang@mail.bnu.edu.cn (C.F.W.); yangk_1988@mail.bnu.edu.cn (K.Y.); 3 Key Laboratory of Radiological Protection and Nuclear Emergency, Chinese Center for Disease Control and Prevention, Haidian District, Beijing 100875, China; E-Mails: fanliyhx@gmail.com (L.F.); fengjiangbin@163.com (J.B.F.); 4 Analytical and Testing Center, Beijing Normal University, Beijing 100875, China; E-Mails: gengzhufeng@bnu.edu.cn (Z.F.G.); dengzw@bnu.edu.cn (Z.W.D.)

**Keywords:** 8-geranyloxypsolaren, 2-methoxy-1-(3-methyl-buten-1-yl)-9*H*-carbazole-3-carbaldehyde, *Clausena lansium*, carbazoles, coumarins, cytotoxic activity

## Abstract

Six compounds were isolated from the stems of *Clausena lansium* (Lour.) Skeels by repeated sillica gel column chromatography. Their chemical structures were elucidated on the basic of physicochemical and spectroscopic data. Among them, 8-geranyloxypsolaren (**3**) and 2-methoxy-1-(3-methyl-buten-1-yl)-9*H*-carbazole-3-carbaldehyde (**6**) were isolated for the first time from this plant. These compounds were screened for cytotoxicity in human cervical cancer (Hela), leukemia (K562), lung cancer (A549), non-small lung carcinoma (H1299) and liver cancer (SMMC-7721). Within the series of cytotoxic tests, compounds **4**–**6** displayed potent cytotoxic activity against H1299 and SMMC-7721, with the IC_50_ values of 6.19 to 26.84 μg/mL.

## 1. Introduction

**Figure 1:**
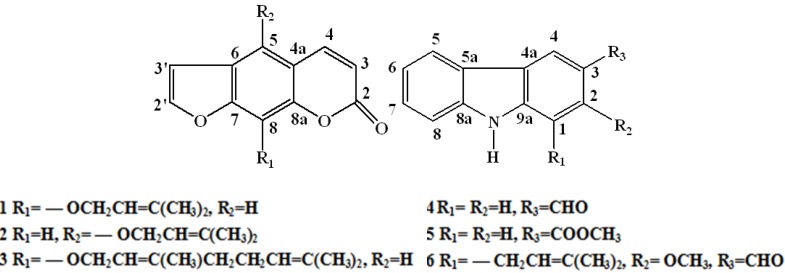
The structures of compounds **1**–**6**.

*Clausena lansium* (Lour.) Skeels (Rutaceae) is widely distributed in the south of China. In Traditional Chinese Medicine, the leaves of *C. lansium* are used for cough, asthma, viral hepatitis, dermatological, and gastrointestinal diseases. Different parts of this plant are used as folk medicines for treatment of acute and chronic viral hepatitis in China [1,2,3]. It was reported that carbazoles and coumarins from *C. lansium* exhibited a variety of bioactivities such as antimicrobial [4,5,6,7,8], anti-inflammatory [9,10,11,12], cytotoxicity [13,14,15,16] and anti-HIV effects [17,18], but no detailed research has been reported about the cytotoxic activity of the compounds isolated from the stems of *C. lansium*. Therefore, as a series of our further research on the stems of *C. lansium*, imperatorin (**1**), isoimperatorin (**2**), 8-geranyloxypsolaren (**3**), 3-formylcarbazole (**4**), methyl carbazole-3-carboxylate (**5**) and 2-methoxy-1-(3-methyl-buten-1-yl)-9*H*-carbazole-3-carbaldehyde (**6**) (Figure 1) were isolated and tested for their cytotoxic activities against Hela, K562, A549, H1299 and SMMC-7721 tumor cell lines *in vitro*. In this paper, we report the isolation, structure elucidation of these six compounds, which include two compounds isolated for the first time from this plant and the cytotoxic activity of compound **3**–**6** against K562, A549, Hela, H1299 and SMMC-7721 tumor cell lines.

## 2. Results and Discussion

Compound **3** was isolated as yellow crystals. The ^1^H-NMR and ^13^C-NMR spectral data, together with a quasi-molecular ion peak in its ESI-MS *m/z*: 339.1599 [M−H]^−^ showed its molecular formula to be C_21_H_22_O_4_. The NMR spectra of compound **3** was similar to those of compound **1**, except that in **1** a isoprenyl was absent at C-5''. In the HMBC spectrum of compound **3**, the isoprenyl position was confirmed by the correlations of H-2''/C-4'', C-5''; H-5''/C-2'', C-3'', C-6'', C-7''. Ultimately, compound 3 was determined to be 8-geranyloxypsolaren. It has been isolated from this plant for the first time.

**Figure 2:**
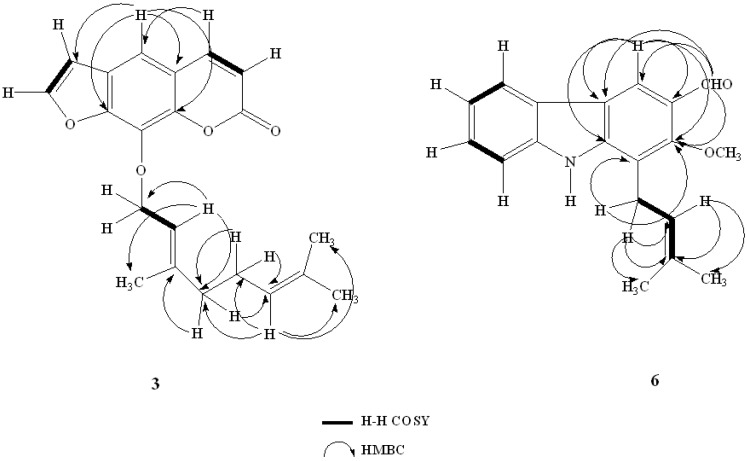
H–H COSY and Key HMBC correlations for compounds **3** and **6**.

Compound **6** was obtained as yellow needles. The ^1^H-NMR and ^13^C-NMR spectral data, together with a quasi-molecular ion peak in its HR-ESI-MS spectrum at *m/z*: 316.1312 [M+Na]^+^ (calc. for C_19_H_19_NO_2_, 293.1415), suggested a molecular formula of C_19_H_19_NO_2_. The ^1^H-NMR spectrum showed characteristic signals for the isoprenyl moiety at δ_H_ 5.26 (d, *J =* 6.5 Hz), 3.97 (d, *J =* 6.5 Hz), 1.87 (s), 1.73 (s), one aldehyde group singlet proton at δ_H_ 10.43 and one methoxy at δ_H_ 3.99. The ^13^C-NMR spectrum displayed one aldehyde group, one methoxy carbon and the isoprenyl group. The above information indicated that the structure of 3-formyl-2-methoxycarbazole *O*-methylmukonal isolated from *Murraya Siamensis* [19] was similar to that of compound **6**, except for the presence of one isoprenyl at C-l. The H-H COSY spectrum exhibited the correlations between H-5/H-6, H-1'/H-2' and H-2'/H-3'. The HMBC spectrum displayed the cross-peaks from H-1' to C-1, C-2, C-2', C-3', and H-2' to C-3', C-4', C-5'. The cross-peaks in HMBC spectrum from H-4 to C-4a, C-9a, C-3, and 3-CHO to C-4, C-4a, C-2 were in accordance with the assignment of 2-methoxy-1-(3-methyl-buten-1-yl)-9*H*-carbazole-3-carbaldehyde (Figure 2). It also has been isolated from this plant for the first time.

The cytotoxicity of six compounds was evaluated against Hela, K562, A549, H1299, SMMC-7721 cancer cell lines, with doxorubicin (DOX) as the positive control. The results are summarized in Table 1. Among the tested compounds, compound **4** showed the most potent cytotoxic activity against K562, H1299 and SMMC-7721 with IC_50_ values of 12.71, 13.23 and 6.19 µg/mL. Compound **5** also exhibited potent cytotoxicity against H1299 and SMMC-7721, with IC_50_ values of 15.77 and 10.69 µg/mL. However, Compound **6** merely exhibited potent cytotoxic effect against SMMC-7721, with an IC_50_ value of 7.61 µg/mL, and showed weak cytotoxic activity against the other cancer cell lines. Up to now there are no reports on the cytotoxicity of compounds **3**–**6** against tumor cell lines. The results suggest that the main cytotoxic activity of the fraction of *C. lansium* might be attributed to 3-formyl carbazole (**4**), methyl carbazole-3-carboxylate (**5**) and 2-methoxy-1-(3-methyl-buten-1-yl)-9*H*-carbazole-3-carbaldehyde (**6**).

**Table 1:** Cytotoxicity of compound **1**–**6** from *Clausena lansium.*

Compound	IC_50_ (µg/mL) ^a^				
	Hela	K562	A549	H1299	SMMC-7721
1	>50	>50	>50	>50	>50
2	>50	>50	>50	>50	>50
3	46.21 ± 2.61	>50	46.27 ± 3.26	>50	25.24 ± 1.86
4	12.71 ± 3.58	20.48 ± 1.76	37.64 ± 1.34	13.23 ± 2.36	6.19 ± 0.59
5	33.08 ± 8.92	26.50 ± 2.12	30.09 ± 1.58	15.77 ± 1.89	10.69 ± 1.83
6	29.85 ± 2.95	23.49 ± 1.85	>50	26.84 ± 1.45	7.61 ± 1.49
DOX ^b^	1.52 ± 0.06	11.04 ± 6.81	2.38 ± 0.22	10.04 ± 0.06	0.77 ± 0.07

^a^ IC_50_ value was the 50% inhibition concentration and calculated from regression lines using five different concentrations in replicate experiments for six time; ^b^ Doxorubicin used in positive control.

## 3. Experimental

### 3.1. General

^1^H- and ^13^C-NMR spectra were recorded on Bruker Avance DRX 500 NMR spectrometer with TMS as the internal standard. ESI-MS and HR-ESI-MS were obtained on a Bruker Q-TOF mass spectrometer. Silica gel (160–200 mesh, 200–300 mesh, Qingdao Marine Chemical Plant, Qingdao, China) used for column chromatography and Sephadex LH-20 were supplied by Amersham Pharmacia Biotech (Beijing, China). Analytical grade solvents were produced by Beijing Chemical Factory (Beijing, China).

### 3.2. Plant Material

The fresh stems (6.0 kg) of *C. lansium* were collected from Yulin, Guangxi Province, China (22.38° N latitude and 106.42° E longitude), September 2011, and identified by Haibo Yin of Liaoning University of Traditional Chinese Medicine. Voucher specimens (BNU-HSL-Dushushan-2011-09-16-015) were deposited at the herbarium (BNU) in the College of Resources Sciences, Beijing Normal University.

### 3.3. Extraction and Isolation

The dried stems (6.0 kg) were extracted under ultrasound three times (each for half an hour) with petroleum ether-ethyl acetate (PE/EtOAc) (12 L). The extract was evaporated *in vacuo* to obtain a crude extract (39.1 g). The suspension was fractionated by silica gel column chromatography (160–200 mesh, 5.5 × 52 cm, 500 g), using a gradient solvent system of CHCl_3_/MeOH (CHCl_3_, 50:1, 30:1, 20:1, 10:1, 1:1 and MeOH) to afford 50 fractions. Silica gel column chromatography (160–200 mesh, 3.5 × 35 cm 160 g) of Fr. 4 (4.18 g) eluting PE/EtOAc (30:1) gave forty subfractions (4.1–4.40). Fr.4.15 (0.37 g) was chromatographed on a gel column (200–300 mesh) eluting with PE/EtOAC (30:1) to give compound **2** (20 mg). Fr. 4.39 (0.25 g) and Fr. 4.29 (0.77 g) were subjected to silica gel column (200–300 mesh) eluting with PE/EtOAC (20:1) to afford compounds **3** (135 mg) and **5** (78 mg). Compound **6** (35 mg) was obtained from Fr. 4.24 (0.56 g) after purification by chromatography on a silica gel column (200–300 mesh). Frs. 8–9 (0.7 g) was subjected to silica gel column (200–300 mesh, 1 × 30 cm, 30 g, PE/EtOAc 15:1), then purified by chromatography on a Sephadex LH-20 column (CHCl_3_/MeOH, 1:1) to give compound **1** (187 mg)and **4** (150 mg).

*Imperatorin* (**1**). White crystals. ESI-MS *m/z*: 271.0926 [M+H]^+^. ^1^H-NMR (CDCl_3_) δ ppm: 7.77 (1H, d, *J* = 9.5 Hz, H-4), 7.70 (1H, d, *J* = 2.0 Hz, H-2'), 7.37 (1H, s, H-5), 6.82 (1H, d, *J* = 2.0 Hz, H-3'), 6.37 (1H, d, *J* = 9.5 Hz, H-3), 5.62 (1H, t, *J* = 7.0 Hz, H-3"), 5.01 (2H, d, *J* = 7.0 Hz, H-2"), 1.74 (3H, s, H-5"), 1.72 (3H, s, H-6"). ^13^C-NMR (CDCl_3_) δ ppm: 160.5 (C-2), 148.5 (C-7), 146.7 (C-2'), 144.4 (C-4), 143.8 (C-8a), 139.7 (C-4''), 131.8 (C-8), 125.9 (C-6), 119.8 (C-3''), 116.5 (C-4a), 114.7 (C-3), 113.5 (C-5), 106.7 (C-3'), 70.2 (C-2''), 25.8 (C-5''), 18.1 (C-6''). The ^1^H- and ^13^C-NMR spectral data were consistent with published data [20,21].

*Isoimperatorin* (**2**). Yellow crystals. ESI-MS *m/z*: 271.0977 [M+H]^+^. ^1^H-NMR (CDCl_3_) δ ppm: 8.19 (1H, d, *J* = 10.0 Hz, H-4), 7.62 (1H, d, *J* = 2.0 Hz, H-2'), 7.18 (1H, s, H-8), 6.98 (1H, d, *J* = 2.0 Hz, H-3'), 6.31 (1H, d, *J* = 9.5 Hz, H-3), 5.57 (1H, t, *J* = 6.5 Hz, H-3"), 4.94 (2H, d, *J* = 7 Hz, H-2"), 1.83 (3H, s, H-5"), 1.72 (3H, s, H-6").^13^C-NMR (CDCl_3_) δ ppm: 161.4 (C-2), 158.7 (C-7), 153.0 (C-8a), 149.1 (C-5), 144.9 (C-2'), 140.1 (C-4"), 139.6 (C-4), 119.4 (C-3"), 116.7 (C-6), 112.6 (C-3), 107.6 (C-4a), 105.0 (C-3'), 94.3 (C-8), 69.8 (C-2"), 26.4 (C-5''), 18.1 (C-6''). The above data were consistent with the literature data [20,21].

*8-Geranyloxypsolaren* (**3**). Yellow crystals. UV (MeOH) λ_max_ (logε) 307.0, 249.0 and 213.0 nm; IR (KBr) ν_max_ 3,134, 3,111, 1,720, 1,706, 1,586 cm^−^^1^, HR-ESI-MS *m/z*: 361.1412 [M+Na]^+^. (calc. for C_21_H_22_O_4_, at *m/z*: 338.1518). ^1^H-NMR (CDCl_3_) δ ppm: 7.76 (1H, d, *J* = 2.0 Hz, H-4), 7.65 (1H, d, *J* = 9.5 Hz, H-2'), 7.32 (1H, s, H-5), 6.79 (1H, d, *J* = 2.0 Hz, H-3'), 6.33 (1H, d, *J* = 9.5 Hz, H-3), 5.58 (1H, t, *J* = 7.0 Hz, H-2''), 5.01 (2H, d, *J* = 7.0 Hz, H-1''), 4.97 (1H, t, H-7''), 1.97 (4H, m, H-5'', H-6''), 1.66 (3H, s, H-4''), 1.61 (3H, s, H-10''),1.53 (3H, s, H-9''). ^13^C-NMR (CDCl_3_) δ ppm: 160.5 (C-2), 148.6 (C-7), 146.6 (C-2'), 144.4 (C-4), 143.8 (C-8a), 143.0 (C-8), 131.8 (C-8''),126.5 (C-3''), 125.9 (C-6), 123.7 (C-7''), 119.4 (C-2''), 116.4 (C-4a), 114.7 (C-3), 113.5 (C-5), 106.7 (C-3'), 70.2 (C-1''), 39.3 (C-5''), 26.3 (C-6''), 25.4 (C-10''), 17.5 (C-9''), 16.3 (C-4'') [22].

*3-Formylcarbazole* (**4**). White crystals. ESI/MS *m/z*: 218.0485 [M+Na]. ^1^H-NMR (CDCl_3_) δ ppm: 10.13 (1H, s, 3-CHO), 8.56 (1H, s, NH), 8.64 (1H, s, H-4), 8.16 (1H, d, *J* = 8 Hz, H-5), 8.01 (1H, d, *J* = 8.5 Hz, H-2), 7.55 (1H, d, *J* = 8.5 Hz, H-1), 7.52 (2H, m, H-8 and H-7), 7.36 (1H, dd, *J* = 8.5 Hz, H-6). ^13^C-NMR (CDCl_3_) δ ppm: 191.9 (3-CHO), 143.3 (C-8a), 140.0 (C-9a), 129.2 (C-3), 127.4 (C-7), 124.0 (C-4a), 123.6 (C-5a), 123.2 (C-4), 120.8 (C-5), 120.7 (C-6), 126.9 (C-2), 111.1 (C-8), 110.9 (C-1). The above data were identical to the literature data [23,24]. 

*Methyl carbazole-3-carboxylate* (**5**). Yellow solid. ESI-MS *m/z*: 226.0924. ^1^H-NMR (CDCl_3_) δ ppm: 8.85 (1H, s, H-4), 8.43 (1H, s, N-H), 8.17–8.14 (2H, m, H-2 and H-5), 7.48–7.49 (2H, m, H-7 and H-8), 7.46 (1H, d, *J* = 8.5 Hz, H-1), 7.34 (1H, m, H-6), 4.07 (3H, s, 3-OCH_3_). ^13^C-NMR (CDCl_3_) δ ppm: 167.9 (C=O), 142.3 (C-8a), 139.4 (C-9a), 127.5 (C-2), 126.6 (C-7), 123.3 (C-4a), 123.1 (C-3), 122.9 (C-4), 121.4 (C-5a), 120.7 (C-5), 120.4 (C-6), 110.9 (C-8), 110.1 (C-1), 51.9 (3-OCH_3_). Its NMR data were in accord with the reported data [25,26,27,28].

*2-Methoxy-1-(3-methyl-buten-1-yl)-9H-carbazole-3-carbaldehyde* (**6**). Yellow needles. HR-ESI-MS *m/z*: 316.1312 [M+Na]^+^ (calcd. for C_19_H_19_NO_2_, 293.1415). ^1^H-NMR (CDCl_3_) δ ppm: 10.43 (1H, s, 3-CHO), 8.52 (1H, s, N-H), 8.47 (1H, s, H-4), 8.12 (1H, d, *J* = 8.0 Hz, H-5), 7.52 (2H, m, H-7 and H-8), 7.32 (1H, m, H-6), 5.26 (1H, t, *J* = 6.5 Hz, H-2'), 3.99 (3H, s, 2-OCH_3_), 3.97 (2H, d, *J* = 5.0 Hz, H-1'), 1.87 (3H, s, H-4'), 1.73 (3H, s, H-5'). ^13^C-NMR (CDCl_3_) δ ppm: 191.8 (3-CHO), 142.9 (C-2), 139.8 (C-8a), 136.9 (C-9a), 133.9 (C-4a), 132.1 (C-3'), 127.9 (C-1), 126.7 (C-7), 124.1 (C-3), 123.8 (C-2'), 123.3 (C-5a), 121.1 (C-4), 120.8 (C-5), 120.7 (C-6), 111.2 (C-8), 61.6 (2-OCH_3_), 25.7 (C-5'), 24.1(C-1'), 18.2 (C-4'). 

### 3.4. Cytotoxicity Assay

The cytotoxicity of compound **1**–**6** were determined by the CCK-8 assay. Hela (cervical cancer), K562 (leukemia), A549 (lung cancer), H1299 (non-smalllung carcinoma), SMMC-7721(liver cancer) were purchased from the Chinese Academy of Medical Sciences (Beijing, China). Doxorubicin (DOX, adriamycin, Actavis Italy S.p.A., Beijing, China) was the positive control. All cells were grown and maintained in RPMI 1640 (Sigma, St. Louis, MO, USA) medium supplemented with 10% fetal bovine serum (Grand Island, NY, USA), 100 IU/mL penicillin (Flow Lab, Beijing, China) and 100 μg/mL streptomycin (Flow Lab, Beijing, China) at 37 °C, 5% CO_2_ and 90% humidity. Cancer cells were seeded in the growth medium (100 µL) into 96 well microtiter plate (5 × 10^3^ cells per each well). After 4–6 h preincubation in the incubator (Forma Series ΙΙ Water Jacket) to allow cellular attachment, various concentrations of test solution were added and cells were incubated for 36 h. At the end of the incubation, CCK-8 reagent (Cell Counting Kit-8, Dojindo, Kumamoto, Japan, 10 μL) was added into each well followed by further incubation for 2 h. The optical density (OD) was measured at 450 nm using a multiscan microplate reader (Thermo, Shanghai, China). Each determination represented the average mean of six replicates. The half maximal growth inhibitory concentration (IC_50_) value was calculated the line equation of the dose-dependent curve of each compound.

## 4. Conclusions

Phytochemical investigation of the PE/EtOAc extract of *C. lansium* led to two compounds isolated for the first time from this plant, 8-geranyloxypsolaren (**3**) and 2-methoxy-1-(3-methyl-buten-1-yl)-9*H*-carbazole-3-carbaldehyde (**6**) and four known compounds, imperatorin (**1**), isoimperatorin (**2**), 3-formylcarbazole (**4**) and methyl carbazole-3-carboxylate (**5**). All the compounds were tested for their *in vitro* cytotoxic activities against Hela, K562, A549, H1299 and SMMC-7721 tumor cell lines. Compound **4** showed the most potent cytotoxic activity against K562, H1299 and SMMC-7721, while compound **5** exhibited potent cytotoxicity against H1299 and SMMC-7721 and compound **6** exhibited potent cytotoxic effects only against SMMC-7721.
